# Rectus Femoris Neuromechanical Responses to Exercise-Induced 3% Body Mass Loss by Baseline Hydration Status: A Randomized Group Comparison

**DOI:** 10.3390/nu18122015

**Published:** 2026-06-21

**Authors:** Karol Skotniczny, Artur Terbalyan, Paweł Linek, Jakub Chycki

**Affiliations:** 1 Institute of Sports Science, Academy of Physical Education, 40-065 Katowice, Poland; a.terbalyan@awf.katowice.pl (A.T.); j.chycki@awf.katowice.pl (J.C.); 2 Institute of Physioterapy and Health Sciences, Academy of Physical Education, 40-065 Katowice, Poland

**Keywords:** dehydration, elasticity, stiffness, shear modulus, twitch-kinetics, baseline hydration status

## Abstract

Background: Acute dehydration impairs performance, but its effects on resting neuromuscular and tissue mechanics are unclear. We tested whether baseline hydration status and exercise-induced sweat loss alter the resting neuromechanical phenotype of the rectus femoris (RF) as well as skin, subcutaneous tissue (subQ), and fascia overlying the RF. Methods: Thirty physically active men were randomized to hydration guidance (EXP) or habitual intake (CON). Hydration was verified weekly using first-morning urine specific gravity (USG), with targets of USG < 1.018 (EXP) and USG > 1.018 (CON). Participants performed continuous cycling at 50% maximal power output (Wmax) until ~3% body mass loss. Shear-wave elastography quantified tissue shear modulus (kPa), and tensiomyography assessed RF twitch-derived contractile properties (Dm, Tc, Tr) before and immediately after exercise. SWE data were analyzed using mixed design repeated-measures ANOVA; TMG outcomes were analyzed using non-parametric tests. Results: Baseline measures did not differ between groups. No significant group, time, or interaction effects were observed for RF muscle, skin, or subQ shear modulus. In contrast, fascia shear modulus showed a significant time effect, while TMG outcomes did not change significantly from pre- to post-exercise (all *p* > 0.05). Deep fascia showed a significant main effect of time, with decreased shear modulus post-exercise (F(1, 21) = 5.06, *p* = 0.035, η^2^p = 0.194; Δ = 1.25 kPa; d = 0.41; 95% CI [0.04, 0.78]), independent of hydration group. Conclusions: Under moderate-intensity cycling with approximately 3% body mass loss, we did not detect significant hydration-group differences or significant pre–post changes in resting RF twitch-derived contractile properties or in RF muscle, skin, and subQ shear modulus. Fascia shear modulus decreased after exercise irrespective of hydration group. These findings should be interpreted cautiously: the study was underpowered to detect small effects, and the fascial finding emerged from an exploratory, layer-specific analysis without correction for multiple comparisons. It should therefore be regarded as preliminary and hypothesis-generating, requiring confirmation in adequately powered, pre-registered studies.

## 1. Introduction

Acute dehydration—particularly that induced by continuous exercise or thermal stress—impairs physical performance, yet its resting neuromuscular and mechanical correlates remain poorly defined. Some studies have shown that acute dehydration of ≥2% body mass reduces maximal strength during slow isokinetic and isometric contractions, with minimal effects on brief, explosive efforts [1,2]. However, non-invasive assessments linking baseline hydration status and exercise-induced sweat loss to skeletal muscle contractile and mechanical properties are scarce. Such information may help characterize resting neuromechanical responses to hydration status and exercise-induced fluid loss and inform the design of future studies linking these measures to physical-performance outcomes.

Tensiomyography (TMG) is a non-invasive method for assessing skeletal muscle contractile properties by recording the radial displacement of the muscle belly in response to a single electrical stimulus [3]. From the displacement-time waveform, standard TMG parameters are derived, including maximal radial displacement (Dm), contraction time (Tc), delay time (Td), sustain time (Ts), and half-relaxation time (Tr) [4]. Field study with combat sports athletes using TMG with acute dehydration have suggested that lower total body water is associated with slower contractile kinetics. Elite wrestlers classified as less hydrated (total body water <60%) exhibited systematically longer Tc in thigh muscles compared with better-hydrated peers [5]. Evidence from laboratory studies is mixed. Evetovich et al. found no dehydration-related changes in torque, electromyographic (EMG), or mechanomyographic (MMG) outcomes during isometric and isokinetic contractions [6]. In contrast, Zubac et al. reported reduced force output and impaired voluntary activation following rapid weight loss, assessed using maximal voluntary contractions and the interpolated twitch technique [7].

Shear-wave elastography (SWE) is an ultrasound technique quantifies tissue mechanical properties by simply assessing shear modulus. Shear modulus refers to tissue elasticity. Some studies have confirmed that muscle elasticity measured by SWE is strongly related to passive and active force generated by the muscles [8,9,10]. Thus, SWE is useful for obtaining selective information on the mechanical properties of muscles in both athletes and non-athletes. SWE is also used to assess other structures, such as skin or subQ [11,12]. This passive mechanical state is plausibly sensitive to fluid balance and the microstructural consequences of sweat-induced dehydration. A recent study using SWE has suggested that mechanical properties of muscle respond to prolonged exercise and fluid shifts in a context-dependent manner: quadriceps elasticity tend to decrease immediately after marathon-type running [13], may increase across multi-day ultra-endurance efforts [14], and appears relatively unchanged after extracellular fluid removal during hemodialysis [15]. Importantly, complementary evidence from a strain elastography study has indicated that 20 min of water-walking can decrease RF and medial gastrocnemius muscles` “hardness” immediately post-exercise, whereas matched land-walking shows little or no change, consistent with hydrostatic-pressure fluid-shift (oedema-reduction) effects [16]. Taking all these findings together, it could be indicated that SWE can detect hydration- and sweat-loss–related alterations in muscle properties.

The present study shifts the focus from performance outcomes to the resting neuromechanical phenotype of skeletal muscle as a function of hydration. Specifically, we aimed to determine whether baseline hydration status and acute exercise-induced sweat loss are associated with changes in the contractile properties of the rectus femoris (RF), assessed using tensiomyography (TMG), and in the shear modulus of the RF muscle and the overlying skin, subcutaneous tissue (subQ), and fascia, assessed using shear-wave elastography (SWE).

Our hypotheses and exploratory aims were defined as follows. First, for TMG-derived outcomes, we hypothesized that optimally hydrated individuals would exhibit a more favorable resting contractile profile, reflected by shorter Tc and Tr and greater Dm. Second, given the limited evidence regarding hydration-related differences in tissue shear modulus, the SWE assessment of the RF muscle, skin, subQ, and fascia was treated as exploratory. Third, following acute exercise-induced dehydration corresponding to approximately 3% body mass loss, we hypothesized that individuals with poorer baseline hydration would exhibit greater adverse changes in TMG-derived outcomes and more pronounced changes in RF muscle and fascia shear modulus. In contrast, we did not expect substantial changes in skin or subQ shear modulus because these layers were expected to be less responsive to short-term exercise-induced fluid shifts. TMG and SWE were considered complementary but non-redundant methods for characterizing the resting neuromechanical response to the experimental protocol.

## 2. Materials and Methods

### 2.1. Participants

**Figure 1:**
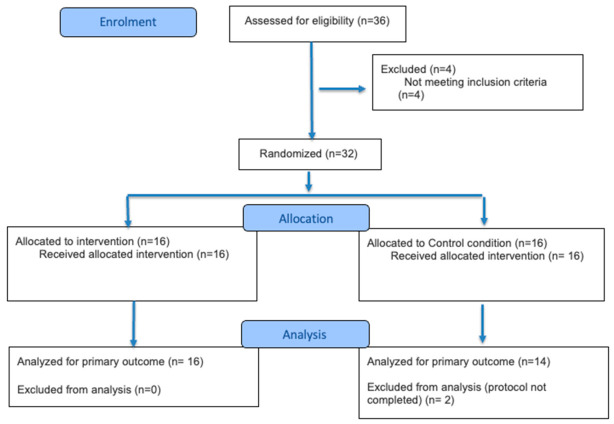
CONSORT Flow Diagram.

Thirty-six healthy, physically active men were assessed for eligibility during a dedicated qualification visit conducted by qualified medical personnel, including a physician. The screening procedure included a structured medical interview, verification of current and past medical history, assessment of medication use, resting electrocardiography (ECG), and confirmation of the absence of medical contraindications to maximal exercise testing and moderate-intensity cycling. Body composition was also assessed to verify non-obese status. Four individuals did not meet the inclusion criteria and were excluded. The remaining 32 participants were randomized to the experimental (EXP; *n* = 16) or control (CON; *n* = 16) condition. Two participants from the CON group did not complete the protocol and were excluded from the final analysis. Consequently, data from 30 participants were analyzed (EXP: *n* = 16; CON: *n* = 14).Results are illustrated in Figure 1.

The inclusion criteria were as follows: male sex, age between 25 and 60 years, regular recreational physical activity, fat mass below 30% of total body mass, absence of acute disease at the time of qualification, no physician-identified contraindications to exercise testing, and provision of written informed consent. Participants were also required to comply with the study procedures, including pre-trial nutritional and hydration instructions, repeated first-morning urine sampling, and exercise testing. The age range of 25–60 years was defined a priori as a pragmatic eligibility criterion intended to limit heterogeneity related to age-associated differences in fluid-balance regulation, thermoregulatory responses to exercise, and skeletal muscle mechanical properties. The study was not designed or powered to evaluate age as an effect modifier, and this restriction should be considered when generalising the findings.

Exclusion criteria comprised any clinically relevant abnormality identified during the medical interview or resting ECG that, in the physician’s opinion, could increase the risk associated with exercise testing; uncontrolled arterial hypertension; unstable coronary artery disease or symptoms suggestive of cardiovascular disease; clinically significant cardiac arrhythmias, conduction abnormalities, or an implanted pacemaker; hepatic or renal disease; acute infection or inflammatory illness; neurological or musculoskeletal disorders limiting safe cycling exercise; and the use of medications or supplements likely to substantially affect fluid balance, neuromuscular function, or exercise tolerance. Participants were also excluded if they were unable to comply with the hydration or nutritional procedures, failed to meet the required hydration status criteria during the qualification phase, or refused or withdrew informed consent.

Hydration status was additionally verified using first-morning urine specific gravity (USG), measured on three occasions separated by at least one week. These measurements served as the hydration-related biochemical screening criterion for proceeding to the experimental intervention and for confirming group-specific hydration status. Participants allocated to the experimental condition were required to achieve USG values < 1.018, whereas participants allocated to the control condition were required to present USG values > 1.018. These criteria were used to distinguish lower-risk and higher-risk habitual hydration profiles rather than to diagnose dehydration. The threshold of USG = 1.018 was not intended as a universal diagnostic cut-off or a formal athlete-specific adjustment; it was defined a priori as a conservative, study-specific operational criterion to identify participants at increased risk of suboptimal habitual hydration before an additional, uncompensated exercise-induced fluid loss of approximately 3% body mass. Because classification was based on repeated first-morning measurements rather than a single urine sample, the influence of day-to-day variability was reduced. Baseline characteristics of subjects are presented in Table 1.

### 2.2. Study Design

To verify the effects of baseline hydration status and exercise-induced sweat loss on neuromuscular and mechanical properties of the RF muscle, a single series of experimental procedures was conducted, preceded by a qualification phase. Participants were randomized to an experimental (EXP) or control (CON) condition. The EXP group received precise, individualized guidance on daily hydration and fluid replacement during habitual physical activity, whereas the CON group was instructed to maintain usual fluid-intake practices. Hydration status was subsequently verified at weekly intervals by analyzing first-morning urine for specific gravity (USG). Classification was based on the mean value calculated from the three measurements, with the additional requirement that at least two of the three individual measurements met the relevant criterion: USG < 1.018 for the optimally hydrated group or USG > 1.018 for the suboptimally hydrated group.

This allowed us to determine the optimal hydration in the EXP group (USG < 1.018) and indicating suboptimal habitual hydration in the control group (USG > 1.018)—both prerequisites for conducting the target research intervention. All participants received oral and written information about the study, were advised of their right to withdraw at any time, and signed written informed consent. The protocol was approved by the Research Ethics Committee of the Academy of Physical Education in Katowice, Poland (No. 3-X/2023).

The study began with a qualification phase during which a series of assessments was performed to evaluate body mass and composition, along with an exercise test to determine aerobic capacity and maximal intensity. These data served to prescribe relative exercise intensities for the main research intervention. Before morphostructural and exercise testing, participants underwent physician-supervised screening to verify inclusion and exclusion criteria. This included a medical history interview, resting ECG, and verification of clinical eligibility and contraindications to maximal exercise testing. Only participants who met the clinical safety criteria and subsequently fulfilled the group-specific hydration criteria based on repeated first-morning USG measurements were allowed to proceed to the main experimental intervention.

Aerobic capacity was assessed with a ramp test (20 W·min^−1^) performed on an Excalibur Sport cycle ergometer (Lode B.V., Groningen, the Netherlands). Testing began at 40 W and proceeded with a continuously increasing work rate (resistance increment 0.33 W·s^−1^) at a supervised cadence of 70–80 rev·min^−1^, continuing to volitional exhaustion or until cadence could no longer be maintained. The presence of a VO_2_ plateau confirmed VO_2_max despite further workload increments (ΔVO_2_ < 150 mL·min^−1^) and a respiratory exchange ratio (RER) > 1.10. Heart rate (HR), minute ventilation (V̇E), oxygen uptake (V̇O_2_), and carbon dioxide output (V̇CO_2_) were recorded continuously at rest and during exercise using a MetaLyzer 3B-2R metabolic analyzer (CORTEX Biophysik GmbH, Leipzig, Germany). Maximal power output (Wmax) was then determined for each participant to prescribe relative exercise intensity.

The experimental phase followed qualification and comprised RF muscle, skin, subQ tissue, fascia SWE and TMG measurements administered before and after an exercise intervention that induced dehydration corresponding to 3% of body mass. Both measurements (SWE and TMG) were performed on the same part of the skin. The operators performing the SWE and TMG assessments were blinded to group allocation and were not informed of the participants’ hydration status classification. However, because the PRE and POST measurements were performed on the same day, immediately before and after the exercise intervention, blinding of the operators to the assessment time point was not feasible.

**Figure 2:**
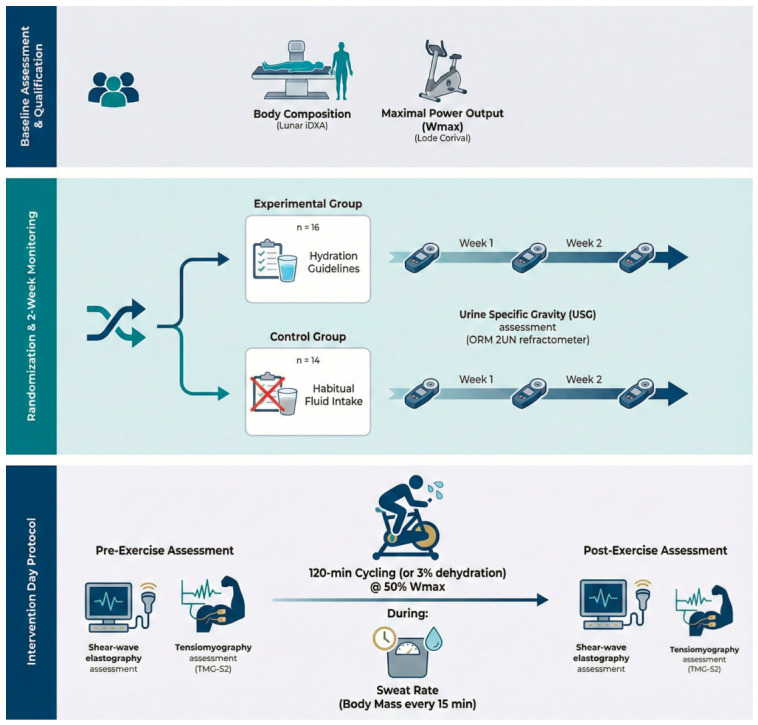
Study Methodology Flowchart.

The exercise bout was performed on a cycle ergometer at a relative intensity of 50% Wmax, referenced to the maximal power test obtained during qualification. Before testing, participants were instructed to consume 300 mL of table water upon waking and to eat a standardized breakfast. Upon arrival at the laboratory, hydration status was re-evaluated by determining the USG of a urine sample. Following the exercise intervention, elastography and tensiomyography assessments were repeated in the same selected muscle groups that had been evaluated prior to the intervention. Flowcharts are illustrated in Figure 2.

### 2.3. Body Composition Assessment (DXA)

Whole-body analysis of body composition to characterize participants of the study was performed with dual-energy X-ray absorptiometry (DXA) using the Lunar iDXA Advance system (GE Healthcare Lunar, Madison, WI, USA). The scanner applies dual photon energies of approximately 39 and 71 keV derived from a 100 kV source with K-edge filtration. Body scans were carried out with subjects positioned supine as per the manufacturer’s protocol. The outputs included body mass (BM, kg), lean body mass (LBM, kg), fat mass (FM, kg), fat percentage (FM, %), and bone mineral density (BMD, g/cm^2^), all computed by the device software. When standardized scanning and positioning protocols are followed, whole-body DXA shows strong short-term precision, and in physically active adults, repositioning introduces only negligible error in whole-body measures—supporting robust test–retest reliability under controlled conditions [17].

### 2.4. Elastography Assessment

An Aixplorer ultrasound scanner (product version 12.2.0, software version 12.2.0.808; Supersonic Imagine, Aix-en-Provence, France) with a linear probe (2–10 MHz; SuperLinear 10-2, Vermon, Tours, France) was used to assess the shear modulus of tissues (RF muscle, skin, subQ, fascia). The measurements were localised on the anterior part of the thigh and expressed in kilopascals (kPa). SWE scans were collected from the anterior region of the right thigh. Each participant lay in a supine position with lower limbs extended and fully relaxed. Participants remained in this position for at least 3 min before measurements. This interval was standardized across all participants to allow tissue relaxation and ensure consistent probe placement. Muscle relaxation was confirmed by visual inspection before each measurement.

**Figure 3:**
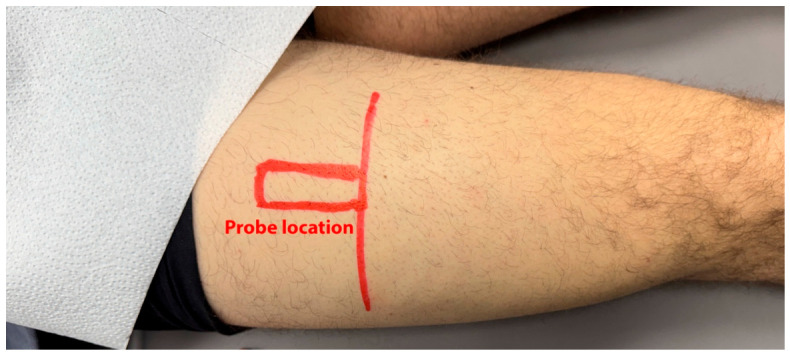
Standardised probe position. The probe was placed at approximately 50% of the distance between the anterior inferior iliac spine and the base of the patella, over the mid-belly of the rectus femoris.

Prior to the SWE measurements, the probe location was determined based on a prior B-mode ultrasound identification of the RF muscle. Finally, the probe was positioned in the middle of the thigh between the anterior inferior iliac spine and the base of the patella (approximately 50% of the distance between landmarks) at the mid-belly of the RF (evaluated using a B-mode scan with the probe in a transverse orientation). The probe was always oriented longitudinally relative to the RF muscle and kept perpendicular to the skin surface, with almost no probe movement duringelastogram stabilization. Before the first scan, the probe position was marked on the skin using a water-resistant skin marker. The same mark remained visible throughout the protocol and was used to reposition the probe during the post-exercise assessment (Figure 3). This allowed measurements to be taken from exactly the same place after the experiment. Before each scan collection, a sufficient amount of hypoallergenic transduction gel was applied to reduce the load on the skin, as pressure significantly affects the muscle’s shear modulus. SWE images were taken 3 times by a physiotherapist, and the mean of the 3 measurements was used for further analysis.

Shear modulus of the skin, subQ tissue, fascia, and RF muscle was calculated from stored images. The elastography acquisition window was approximately 2 cm wide, with its height adapted to the participant’s anatomy and target-tissue depth. For each tissue, three circular Q-Box ROIs were placed entirely within the structure, with ROI diameter adjusted to tissue thickness. The mean value from the three ROIs was used for analysis. Operators were blinded to group allocation and hydration status classification but, because the PRE and POST scans were obtained sequentially on the same day, could not be blinded to the assessment time point. Formal inter- and intra-operator reliability (e.g., intraclass correlation coefficients) was not re-established in the present sample. However, the standardized acquisition protocol followed previously validated procedures, and the reliability of SWE-derived shear modulus of skeletal muscle has been documented for comparable settings and equipment [18]. The absence of sample-specific reliability metrics is acknowledged as a limitation.

### 2.5. Neuromuscular Assessment

**Figure 4:**
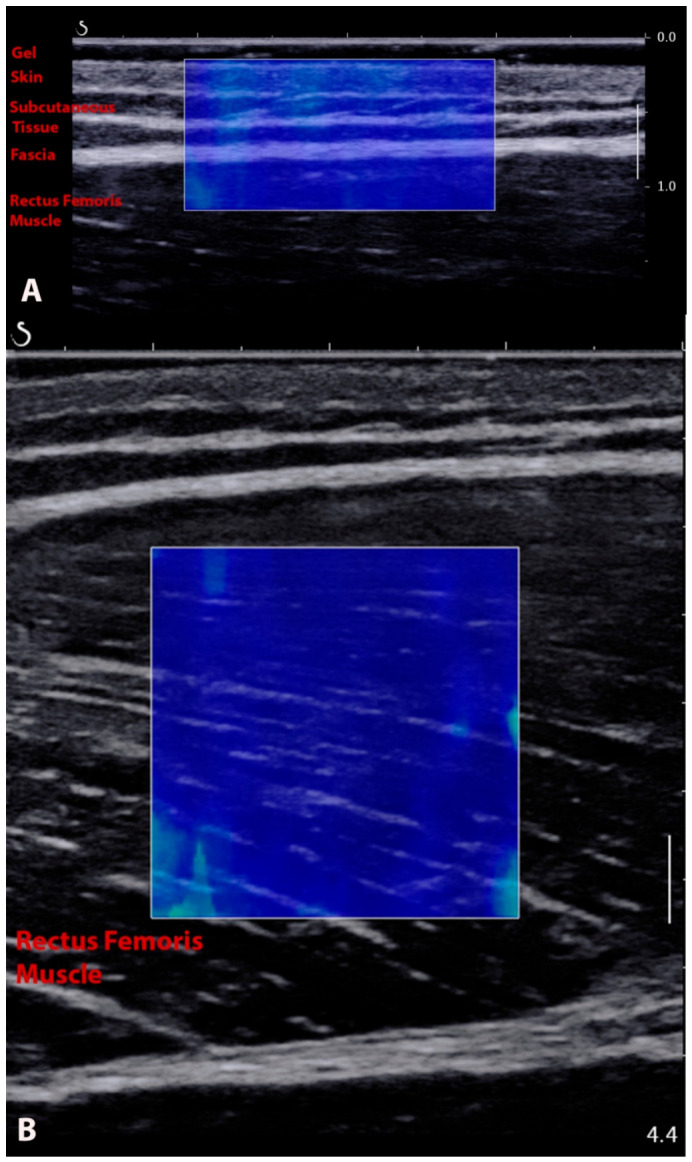
SWE scans showing skin, subQ and fascia above the RF muscle (**A**) and the RF muscle (**B**) for shear modulus measurement. The blue rectangular area represents the SWE acquisition box with the color-coded elastogram superimposed on the B-mode ultrasound image.

TMG was employed to characterise muscle contractile properties—Tc, Tr and Dm. All assessments were performed by a certified TMG operator. Measurements utilised an electrical stimulator (TTMAMG-S2), TMG-OK 3.0 software, and a displacement sensor with a prefixed tip tension of 0.17 N/m^2^ (TMG-BMC, Ljubljana, Slovenia). Sensor placement followed the procedure described by Jones et al. (2017) [19]. Both sensor and electrode sites were marked with a semi-permanent pen to enable exact repositioning for reliability testing. In practice, the TMG sensor was positioned at the same location as the ultrasound probe (Figure 4). For the RF muscle, participants were positioned supine with a triangular wedge foam cushion (TMG-BMC) placed behind the knee of the dominant leg. In this position, the knee angle was fixed at 120° (with 180° denoting full extension). The initial probe site for the RF was set at the midpoint between the muscle’s lateral and medial borders. This was assessed using B-mode ultrasound. Throughout testing, the sensor tip was oriented perpendicular to the muscle belly. Two 5 × 5 cm self-adhesive electrodes (YT5050, YT Healthcare, Shaoxing, China) were applied over the RF. Electrical stimulation consisted of single 1 ms monophasic square-wave pulses delivered using a constant-current stimulator. Three stimuli were applied at progressively increasing current amplitudes of 50, 75, and 100 mA. A 10 s rest interval was provided between consecutive stimuli. For each assessment, the trial producing the highest elicited maximal radial displacement (Dm) was selected for analysis. This standardized incremental protocol was used to identify the greatest contractile response within the predefined stimulation range while limiting the potential effects of repeated stimulation. Ten-second rest intervals were provided between successive stimulations. This interval was standardized across all participants and assessments to reduce, although not necessarily eliminate, potential carryover effects associated with repeated stimulation [20]. TMG assessments were performed prior to the dehydration-inducing exercise intervention to establish the baseline neuromuscular activity profile and to compare individuals who were optimally versus less optimally hydrated. A repeat evaluation was conducted following the intervention, which resulted in uncompensated water loss.

### 2.6. Hydration Status

Hydration status was assessed via USG using a Kern ORM 1SU refractometer, which was calibrated with distilled water according to the manufacturer’s guidance. Each participant underwent three measurements spaced at no less than one-week intervals. Initiation of the experimental intervention was permitted only when the hydration criteria specified by the randomized allocation—EXP or CON—were satisfied. Agreement-focused studies suggest that urine specific gravity (USG) values obtained via refractometry align closely between different instruments, reinforcing their validity for screening applications [21,22].

### 2.7. Nutrition and Hydration Guidelines

After the qualifying phase, participants in the EXP group were given structured hydration guidelines. These stipulated a daily fluid intake of 2.5 L, in accordance with current recommendations for adult men [23] (EFSA Panel on Dietetic Products, Nutrition and Allergies, 2010). Pre-exercise hydration protocols were defined as follows: an intake of 5–7 mL per kg of body mass 4 h prior to exercise and an additional 3–5 mL per kg two hours before exercise if urine colour indicated hypohydration, as outlined by Sawka et al. (2007) [24]. During exercise, fluid ingestion was prescribed at 200–300 mL every 15 min [25]. Post-exercise rehydration was tailored to each individual according to changes in body mass, with participants instructed to restore 120–150% of the fluid deficit calculated from pre- and post-exercise body mass measurements.

Pre-trial nutritional intake was standardised. Two hours before testing, participants consumed a meal providing approximately 4.5 kcal per kg of body mass, consisting of 65% carbohydrates, 25% fats, and 15% proteins. In addition, a fluid bolus of 3–5 mL/kg was administered two hours before the start of the trial. The consumption of any additional beverages was prohibited until the end of intervention protocol.

### 2.8. Statistical Analysis

Data are presented as mean +/− standard deviation (SD) and 95% confidence intervals (CI), or as median (interquartile range [IQR], minimum–maximum) depending on normality of data distribution, assessed using the Shapiro–Wilk test. For variables meeting parametric assumptions, homogeneity of variance was verified using Levene’s test. Baseline characteristics between the experimental and control groups were compared using independent samples t-tests for normally distributed data or Mann–Whitney U tests for non-normally distributed data.

To maintain the direct physiological interpretation of the raw TMG units, data transformations were not applied. Prior to analysis, data were screened for outliers using visual inspection of boxplots; all recorded values were deemed physiologically plausible and were retained in the final analysis to preserve natural sample variance.

For SWE variables, which followed a normal distribution, a mixed-model two-way ANOVA was applied, with group (CON, EXP) modelled as a between-subjects factor and time (PRE, POST) as a within-subjects (repeated-measures) factor. Partial eta squared (η^2^p) was reported as the effect size for ANOVA, with values of 0.01, 0.06, and 0.14 representing small, medium, and large effects, respectively [26]. Pairwise comparisons were conducted using Tukey’s post hoc test. For significant findings, Cohen’s d effect size was calculated and interpreted as small (0.2), medium (0.5), or large (0.8) [26].

For variables that did not meet parametric assumptions (e.g., TMG parameters), non-parametric procedures were applied. Between-group differences at each time point (pre and post) were examined using the Kruskal–Wallis test, and within-group changes over time (pre vs post) were assessed using the Wilcoxon signed-rank test (Z). For non-parametric comparisons, effect sizes were estimated as the rank-biserial correlation (r), computed from the test statistic and sample size, and interpreted using thresholds of 0.10 (small), 0.30 (medium), and 0.50 (large). All tests were two-tailed, with statistical significance set at alpha = 0.05.

Although an a priori sample size calculation was not performed, a post-study sensitivity analysis was conducted using G*Power software (version 3.1, Heinrich-Heine-Universität, Düsseldorf, Germany) to determine the minimum detectable effect size. For the primary mixed-model ANOVA, given a total sample size of *n* = 30, an alpha level of 0.05, and a statistical power of 0.80, the minimum detectable effect size was medium (effect size f = 0.265, corresponding to η^2^p ≈ 0.066). The study was therefore able to detect medium or larger effects but was underpowered to detect small effects; this constraint is considered further in the Limitations.

Statistical analyses were performed using GraphPad Prism 10 (GraphPad Software, Boston, MA, USA).

## 3. Results

The analysis confirmed that all SWE variables met the assumption of normality (Shapiro–Wilk *p* > 0.05), formally justifying the use of parametric repeated-measures ANOVA. Conversely, all TMG variables (Tc, Tr, and Dm) significantly deviated from a normal distribution (Shapiro–Wilk *p* < 0.05) across multiple group-time combinations. Baseline assessments showed no significant differences between the EXP and CON groups for any of the assessed morphostructural or neuromechanical variables (*p* > 0.05). Basic descriptive statistics for TMG and SWE are shown in Tables S1 and S2 respectively.

### 3.1. Tensiomyography

**Figure 5:**
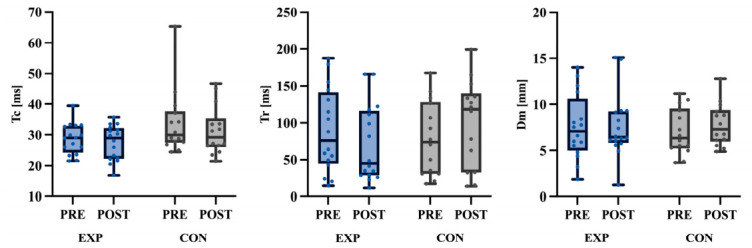
Box-and-whisker plots of tensiomyography-derived variables at pre- and post-assessment. Boxplots show the median (horizontal line), interquartile range (IQR) (box), and minimum–maximum values (whiskers). EXP is shown in blue and CON in grey. Tc = contraction time (ms); Tr = relaxation time (ms); Dm = maximal radial displacement (mm); PRE = pre-intervention; POST = post-intervention; EXP, experimental group; CON = control group.

Across TMG-derived outcomes, no significant within-group changes over time and no between-group differences at either time point were observed. Specifically, Tc remained stable in both groups (EXP: Z = −1.24, *p* = 0.215; CON: Z = −0.97, *p* = 0.331), with no between-group differences at baseline (χ^2^(1) = 2.12, *p* = 0.146) or post-intervention (χ^2^(1) = 1.36, *p* = 0.244). Similarly, Tr did not differ between groups at baseline (χ^2^(1) = 0.209, *p* = 0.647) or post-intervention (χ^2^(1) = 1.453, *p* = 0.228), and no significant time effects were detected within either group (EXP: Z = −1.19, *p* = 0.234; CON: Z = −1.41, *p* = 0.158). Finally, Dm showed no significant changes over time in the EXP or CON group (EXP: Z = −0.21, *p* = 0.836; CON: Z = −1.57, *p* = 0.116), and between-group comparisons were non-significant both pre (χ^2^(1) = 0.125, *p* = 0.724) and post (χ^2^(1) = 0.111, *p* = 0.739). Effect sizes (rank-biserial r) for all non-parametric comparisons were small. For the within-group (pre–post) Wilcoxon tests, r values were Tc, 0.22 (EXP) and 0.18 (CON); Tr, 0.21 (EXP) and 0.27 (CON); and Dm, 0.04 (EXP) and 0.30 (CON). For the between-group (Kruskal–Wallis) comparisons, r ranged from 0.06 to 0.27. Results are illustrated in Figure 5.

### 3.2. Shear-Wave Elastography

For the RF muscle, there was no significant main effect of time (F(1, 27) = 2.64, *p* = 0.116), no significant effect of group (F(1, 27) = 0.002, *p* = 0.965), and no significant time-by-group interaction (F(1, 27) = 0.006, *p* = 0.941). Group-specific means and standard deviations for all SWE-derived variables at the PRE and POST assessments are presented in Supplementary Table S2.

**Figure 6:**
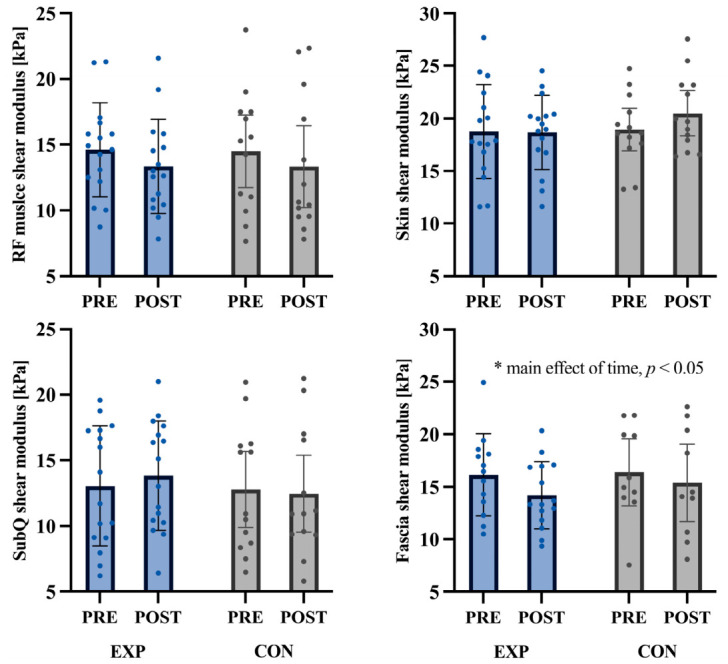
Data are presented as bar charts representing the group means, with error bars indicating the standard deviation (SD). Individual raw data points are overlaid on the bars to illustrate the sample distribution, individual variance, and data spread. Bar charts show shear modulus (kPa) for the skin, subcutaneous tissue, deep fascia, and rectus femoris muscle at the PRE and POST assessments; EXP is shown in blue and CON in grey. EXP = experimental group; CON = control group. * Statistical significance.

Similarly, the properties of the skin remained stable across the protocol, with no significant effects for time (F(1, 27) = 0.74, *p* = 0.397), group (F(1, 27) = 0.80, *p* = 0.380), or their interaction (F(1, 27) = 0.92, *p* = 0.345). SubQ also showed no significant changes related to time (F(1, 27) = 0.18, *p* = 0.675), group (F(1, 27) = 0.25, *p* = 0.619), or the interaction effect (F(1, 27) = 1.04, *p* = 0.317). In contrast, a significant main effect of time was observed for the deep fascia overlying the RF (F(1, 21) = 5.06, *p* = 0.035, η^2^p = 0.194). Subsequent post hoc comparisons confirmed a significant decrease in fascial shear modulus from pre- to post-assessment (∆ = 1.25, *p* = 0.035; d = 0.41; 95% CI [0.04, 0.78]). Because five tissue-layer SWE comparisons were examined without correction for multiple comparisons, this fascia time effect is considered exploratory and hypothesis-generating; it would not remain significant under a conservative Bonferroni-adjusted threshold (α = 0.01 for five SWE variables) and requires confirmation in adequately powered, pre-registered studies. However, no significant main effect of group (F(1, 21) = 0.09, *p* = 0.763) and no significant time-by-group interaction (F(1, 21) = 0.23, *p* = 0.638) were found. Results are illustrated in Figure 6.

## 4. Discussion

The present study investigated whether baseline hydration status and exercise-induced dehydration corresponding to approximately 3% body mass loss were associated with changes in the mechanical properties of the RF muscle and surrounding tissues, as assessed by SWE and TMG. No significant group or group × time effects were observed for RF muscle, skin, or subQ shear modulus or for any TMG-derived parameter. A non-significant tendency toward reduced RF shear modulus was observed after exercise. In contrast, fascia shear modulus showed a significant main effect of time, indicating a post-exercise decrease irrespective of hydration group.

Within the conditions examined, the present study did not detect hydration-dependent differences in resting RF neuromechanical properties. However, these null findings should not be interpreted as evidence that hydration status has no effect on skeletal muscle mechanics or twitch-derived contractile properties. The relatively small sample size and the sensitivity of the applied measurement methods may have limited the detection of subtle effects. Moreover, the significant fascial response cannot be attributed exclusively to dehydration, as exercise-induced mechanical loading and passive changes in tissue pre-strain may also have contributed.

### 4.1. Shear-Wave Elastography in Relation to Hydration

Contemporary acoustoelastic models emphasise that SWE in hydrated tissues reflects both the solid matrix and the fluid phase [27,28]. Static dilation related to perfusion and hydration can modulate shear-wave speed and, consequently, the apparent shear modulus [27,28]. Despite the theoretical sensitivity of muscle tissue to fluid shifts, the present study did not identify hydration-dependent differences in RF muscle shear modulus. Changes over time were similar in both hydration groups, and only a trend-level reduction in shear modulus was observed. Nwawka et al. (2021) reported that direct intramuscular saline injection (5–10 mL) into cadaveric upper-limb muscles decreased SWE-derived stiffness by approximately 12–22 kPa [29]. However, this comparison should be interpreted cautiously. Cadaveric tissue lacks active muscle tone and physiological perfusion, while direct saline injection produces a localized increase in tissue volume. These methodological differences limit the direct comparability of the findings. Under the conditions examined in the present study, resting RF shear modulus did not appear to be sensitive to acute exercise-induced dehydration corresponding to approximately 3% body mass loss in healthy, physically active men.

The direction and magnitude of SWE changes after exercise, as reported in the literature, depend strongly on the loading characteristics. A systematic review has shown that eccentric, muscle-damaging protocols typically increase shear modulus in the hours and days following exercise across multiple muscle groups, especially when assessed at longer muscle lengths [30]. In contrast, endurance events yield more heterogeneous patterns, with different mechanical properties reported, likely reflecting the interplay of fatigue, damage, and fluid shifts [31,32]. For example, during a 330 km mountain ultra-marathon, Andonian et al. (2016) observed a significant decrease in quadriceps shear modulus at race completion with partial recovery 48 h later, interpreted as the consequence of overuse, inflammation and swelling [14]. The modest, non-significant softening trend of the RF muscle in our study is therefore consistent with an endurance-type, submaximal stimulus that induces relatively small mechanical and fluid-related changes compared with high-load eccentric or extreme ultra-endurance protocols.

By contrast, the fascia overlying the RF muscle showed a significant time effect and the largest effect size among all assessed tissues, whereas skin and subQ shear modulus remained relatively unchanged. This layer-specific response is consistent with the emerging view of fascia as a mechanoresponsive and force-transmitting tissue rather than a passive packing structure [33,34,35,36]. The absence of parallel changes in skin and subQ shear modulus, measured within the same acquisition chain, reduces the likelihood that the finding was driven primarily by a generalized technical artefact, such as probe-pressure variation [18,37,38,39]. However, the post-exercise decrease in fascial shear modulus should be interpreted as an exercise-associated finding rather than a dehydration-specific response. Repeated limb movement may have altered fascial pre-strain, tissue loading, or viscoelastic state. Because the study did not include a non-exercise control condition or an exercise condition with fluid replacement, the relative contribution of these mechanical factors cannot be determined.

Taken together, these findings indicate that SWE-detectable changes were limited at the muscle level in the present sample, whereas fascia shear modulus decreased significantly following exercise. This layer-specific response warrants further investigation but should not be interpreted as evidence of a hydration-specific biological adaptation. Changes in fascial pre-strain, viscoelastic relaxation, repeated limb movement, and other exercise-related mechanical factors may also have contributed. Because functional-performance outcomes were not assessed, the practical significance of the observed fascial response remains unknown. Notably, the RF was assessed in a partially shortened position. Testing the muscle at different lengths or during isometric contraction may reveal effects that are not detectable at rest and should be considered in future studies.

### 4.2. Tensiomyography in Relation to Hydration

In contrast to the layer-specific fascial response, TMG indices of the RF muscle (Dm, Tc, Tr) remained statistically unchanged pre- to post-exercise and between hydration groups in the present study, with only a small trend toward prolonged relaxation time (Tr) in the initially less-hydrated group. This pattern suggests that, within the range of mild–moderate dehydration achieved and at rest, exercise and hydration status did not meaningfully alter twitch-derived contractile properties.

Systematic reviews consistently report high relative and acceptable absolute reliability for Dm and Tc, whereas Tr and show much larger coefficients of variation and measurement error, limiting their usefulness for detecting small changes [40]. The experimental work of Piqueras-Sanchiz et al. (2020) further demonstrated that even under controlled conditions, Tr exhibits poor absolute reliability across electrode sizes and pulse durations, while Dm and Tc remain stable and highly reproducible [41]. Given the poor absolute reliability of Tr, the non-significant trend observed in the initially less-hydrated group should be regarded as inconclusive and may reflect measurement variability rather than a physiological slowing of relaxation [39].

Evidence on TMG’s responsiveness to acute exercise-induced fatigue is mixed. Meta-analysis has shown that strenuous or eccentric loading can reduce Dm and modify Tc in the RF and biceps femoris, indicating increased stiffness and altered contractile kinetics after fatigue [42]. At the same time, other work has reported that TMG markers are not consistently sensitive to recovery status or moderate training loads, particularly when muscle damage is limited [43]. Our null TMG findings after submaximal cycling with ~3% body mass loss therefore aligns with the view that TMG detects relatively large perturbations in contractile function (e.g., high-load eccentric exercise, pronounced muscle damage), but may remain unchanged after endurance-type tasks that primarily induce metabolic fatigue and cardiovascular strain [44].

Hydration-specific TMG data are scarce. García et al. reported that elite wrestlers with lower total body water (<60%) exhibited systematically longer Tc and lower Dm in several quadriceps and hamstring muscles compared with better-hydrated peers [5]. However, these findings were obtained in a markedly different physiological context. Pre-competition weight-cutting practices may involve more prolonged or repeated fluid restriction and may be accompanied by sustained alterations in recovery status, extracellular-matrix hydration, and the distribution of water between extracellular and intracellular compartments [45]. By contrast, the present study examined an acute fluid deficit induced during a single bout of moderate-intensity cycling. Acute exercise-induced fluid shifts may be too transient to alter resting RF twitch mechanics immediately after exercise [46]. This distinction provides a plausible explanation for the absence of significant changes in Tc and Dm in the present study. Nevertheless, the comparison should be interpreted cautiously because the studies differed in design, population, and hydration-assessment methods, and the observations reported by García et al. were cross-sectional. Complementary electromyographic studies have shown that dehydration can reduce muscle activation during cycling or fatiguing contractions without clearly altering peripheral contractile markers, supporting the notion that the neuromuscular consequences of acute dehydration may be mediated predominantly by changes in neural drive, cardiovascular strain, and perceived effort rather than by marked deterioration of single-twitch mechanical properties [47,48].

Overall, the present study did not detect significant changes in resting RF twitch-derived contractile properties following submaximal endurance exercise with approximately 3% body mass loss. The small non-significant tendency observed for Tr should not be interpreted biologically because this parameter has limited absolute reliability and no corresponding changes were observed in Dm or Tc. Larger studies designed specifically to compare acute and longer-lasting hydration perturbations and combining TMG with functional and electrophysiological measures are required to determine whether hydration-related alterations in resting twitch mechanics can be detected reliably [2].

### 4.3. Limitations

This study has several limitations that should be considered when interpreting the findings. First, the absence of a true control condition, such as a non-exercise condition or an exercise condition with fluid replacement, limits causal inference regarding the isolated effect of dehydration. Consequently, the observed post-exercise reduction in fascia shear modulus cannot be attributed specifically to dehydration, as repeated muscle activity, mechanical loading, and other exercise-related physiological responses may also have contributed. The present findings should therefore be interpreted as responses observed after exercise accompanied by progressive body-water loss rather than as isolated effects of dehydration.

Second, the sample size was relatively small (*n* = 30) and consisted exclusively of healthy, physically active men. Although an a priori sample size calculation was not feasible due to the logistical constraints of the rigorous, multi-day hydration screening protocol, a post-study sensitivity analysis indicated that the minimum effect size detectable with 80% power was medium (partial eta-squared ≈ 0.066). The study was therefore powered to detect medium or larger effects but was underpowered to detect small effects, several of which were observed (partial eta-squared as low as 0.006); accordingly, the non-significant findings should not be interpreted as evidence of no effect. Combined with the acute dehydration stimulus of approximately 3% body mass loss, this limits generalisability to women, older adults, clinical populations, or individuals exposed to more prolonged or repeated hypohydration.

Third, neuromechanical properties were assessed only at rest, in a single muscle (RF) and joint position, and only immediately before and after the exercise bout. Therefore, no inferences can be made about recovery dynamics or functional performance during exercise.

Moreover, the study relied on a single imaging modality (SWE) and one neuromuscular technique (TMG), each with method-specific constraints. In particular, SWE is sensitive to probe placement, tissue anisotropy, and depth-related signal degradation, whereas certain TMG parameters (especially Tr) exhibit lower absolute reliability than Dm and Tc [40]. These factors may have reduced sensitivity to subtle physiological changes and likely contributed to the observed trend-level but non-significant effects on muscle stiffness and relaxation time. Sample-specific inter- and intra-operator reliability (e.g., intraclass correlation coefficients) was not formally re-established for either method in the present laboratory; instead, standardized, previously validated protocols were used and prior reliability evidence for SWE [18] and for TMG-derived Dm and Tc [40,41] was relied upon. Because absolute reliability is poorer for Tr, the non-significant Tr observations in particular cannot be confidently distinguished from measurement error and should be regarded as inconclusive.

Furthermore, given the highly correlated and mechanobiologically linked nature of the adjacent tissue layers assessed via SWE at the same anatomical site, and the exploratory nature of layer-specific tissue mapping in this hydration paradigm, formal alpha-level adjustments for multiple comparisons (e.g., Bonferroni correction) were not applied. Such strict corrections assume complete variable independence and would severely inflate the risk of Type II errors. A consequence of this analytical choice is an elevated probability that an isolated significant result may reflect a chance finding. Accordingly, the single significant effect (the post-exercise reduction in fascia shear modulus, *p* = 0.035) is explicitly interpreted as exploratory and hypothesis-generating rather than confirmatory. It would not survive a conservative Bonferroni-adjusted threshold (α = 0.01 for five SWE variables) and requires confirmation in a pre-registered study applying a corrected alpha.

Future studies should combine layer-specific SWE and TMG with direct functional measures to determine whether tissue-mechanical changes observed under different exercise and hydration conditions translate into meaningful alterations in performance. A repeated unilateral knee-extension task performed at 85% of maximal voluntary isometric contraction torque until task failure may be particularly informative, as muscular strength-endurance may be more sensitive to an acute fluid deficit of approximately 3% body mass than peak torque alone [48]. A dynamometer-based maximal voluntary isometric knee-extension test could be included as a complementary measure of maximal force production. Both assessments would provide functionally relevant outcomes for the same muscle group examined using SWE and TMG. Additionally, because our measurements were taken with the RF at a relatively short muscle length, future protocols should assess a wider range of joint angles or include an isometric condition to determine whether exercise- or hydration-related effects become more apparent under higher passive or active tension.

## 5. Conclusions

This study did not detect significant hydration-group differences or group × time interactions in resting RF neuromechanical properties following moderate-intensity cycling with approximately 3% body mass loss. No significant changes were observed in RF muscle, skin, or subQ shear modulus or in TMG-derived contractile parameters. A significant post-exercise reduction in fascia shear modulus was observed across both hydration groups. This finding should be interpreted as an exercise-associated response and cannot be attributed specifically to dehydration on the basis of the present study design. Because it emerged from an exploratory, layer-specific analysis without correction for multiple comparisons, it should be considered preliminary and requires replication in pre-registered studies applying a corrected alpha.

Under these experimental conditions, the study did not detect significant hydration-dependent differences in resting RF neuromechanical properties. This does not rule out smaller effects that the present design was underpowered to detect. The observed fascial response warrants further investigation, but its underlying mechanism and functional relevance remain unclear.

Future studies should include larger samples, appropriate control conditions, direct performance outcomes, and measurements across different muscle groups, exercise modalities, and hydration perturbations to determine whether layer-specific mechanical responses translate into meaningful changes in physical performance or injury risk.

## Figures and Tables

**Table 1:** Baseline anthropometric, body composition, and aerobic capacity characteristics of the participants.

Variable	EXP Group (*n* = 16) Mean ± SD (Min–Max)	CON Group (*n* = 14) Mean ± SD (Min–Max)	*p*-Value
Age (years)	33.9 ± 5.9 (25.0–43.0)	35.0 ± 6.4 (27.0–44.0)	0.684
Height (cm)	180.0 ± 6.9 (166.0–190.0)	179.3 ± 6.1 (168.0–191.0)	0.768
Body mass (kg)	81.0 ± 8.8 (65.4–103.5)	80.3 ± 9.3 (68.1–96.1)	0.841
Lean body mass (kg)	65.6 ± 7.9 (52.1–88.8)	62.1 ± 5.2 (53.6–71.6)	0.164
Fat mass (%)	16.4 ± 4.4 (10.1–25.8)	19.7 ± 6.0 (10.5–29.7)	0.094
Bone mineral density (g/cm^2^)	1.34 ± 0.09 (1.13–1.50)	1.36 ± 0.11 (1.20–1.58)	0.633
VO_2_max (ml/kg/min)	57.3 ± 7.6 (48.0–67.0)	55.8 ± 3.9 (52.0–60.0)	0.718

Data are presented as mean ± standard deviation (min–max). EXP = experimental group; CON = control group; *p*-values derived from independent samples t-tests. Body composition variables (lean body mass, fat mass, bone mineral density) were acquired via dual-energy X-ray absorptiometry (DXA). VO_2_max = maximal oxygen uptake. *p*-value indicates the level of statistical significance.

## Data Availability

The data are available from the corresponding author upon reasonable request.

## Supplementary Materials

The following supporting information can be downloaded at: https://www.mdpi.com/article/10.3390/nu18122015/s1, Table S1: TMG-derived variables before and after the intervention, Table S2: SWE-derived variables before and after the intervention.

## Abbreviations

The following abbreviations are used in this manuscript:

ANOVA	Analysis of variance
BM	Body mass
BMD	Bone mass density
CI	Confidence interval
CON	Control group
Dm	Maximal radial displacement
DXA	dual-energy X-ray absorptiometry
EFSA	European Food Safety Authority
EXP	Experimental group
FM	Fat mass
HR	Heart rate
IQR	interquartile range
LBM	Lean body mass
POST	Post intervention
PRE	Pre intervention
RER	Respiratory exchange ratio
RF	Rectus femoris
SD	Standard deviation
SubQ	Subcutaneous tissue
SWE	Shear-wave elastography
Tc	Contraction time
Td	Delay time
TMG	Tensiomyography
Tr	Half-relaxation time
Ts	Sustain time
USG	Urine specific gravity
VO_2_max	Maximal oxygen uptake
V̇O_2_	Oxygen uptake
Wmax	Maximal power output
η^2^p	Partial eta squared
χ^2^	Chi-square statistic
B-mode	Brightness mode (ultrasound imaging)
kcal	Kilocalorie
keV	Kilo-electron volt
kPa	Kilopascal
kV	Kilovolt
mA	Milliampere
mL	Millilitre
ms	Millisecond
µGy	Microgray
µSv	Microsievert
