# Supplementary material for: Rectus Femoris Neuromechanical Responses to Exercise-Induced 3% Body Mass Loss by Baseline Hydration Status: A Randomized Group Comparison
Source: Nutrients. 2026 Jun 21;18(12):2015. doi: 10.3390/nu18122015 (PMC13305235; doi:10.3390/nu18122015)
Supplement: Supplementary file 1 [file nutrients-18-02015-s001.zip › nutrients-4356690-supplementary.pdf]

Table S1. TMG-derived variables before and after the intervention

Variable	PRE		POST	
	CON	EXP	CON	EXP
Tc (ms)	29.91 (8.75, 24.41–65.50)	28.98 (7.44, 21.57–39.40)	29.20 (7.13, 21.47–46.70)	28.91 (9.52, 16.90–35.80)
Tr (ms)	73.73 (89.24, 17.12–167.80)	75.74 (86.59, 14.51–187.40)	118.29 (94.49, 13.89–199.40)	45.05 (84.20, 11.62–166.10)
Dm (mm)	6.34 (3.82, 3.70–11.20)	7.08 (3.82, 1.86–14.00)	7.33 (2.99, 4.87–12.80)	6.45 (3.22, 1.25–15.10)

Data are presented as median (IQR, minimum–maximum). Tc = contraction time; Tr = relaxation time; Dm = maximal radial displacement; CON = control group; EXP = experimental group; PRE = pre-intervention; POST = post-intervention.

Table S2. SWE-derived variables before and after the intervention

Variable	PRE		POST	
	**CON**	**EXP**	**CON**	**EXP**
Skin (kPa)	18.9±3.35 (16.9–21.0)	18.8±4.48 (16.4–21.1)	20.5±3.56 (18.4–22.7)	18.7±3.52 (16.8–20.5)
Subcutaneous Fat (kPa)	12.8±4.81 (9.9–15.7)	13.0±4.57 (10.6–15.5)	12.5±4.84 (9.5–15.4)	13.8±4.16 (11.6–16.1)
Deep Fascia (kPa)	16.4±4.48 (13.2–19.6)	16.1±3.90 (13.8–18.5)	15.4±5.14 (11.7–19.1)	14.2±3.18 (12.4–16.0)
Muscle (kPa)	15.3±3.78 (12.6–18.0)	15.7±3.63 (13.6–17.9)	13.3±5.16 (10.2–16.5)	13.4±3.60 (11.4–15.3)

Data are presented as Mean (SD) [95% Confidence Interval]. CON = control group; EXP = experimental group; PRE = pre-intervention; POST = post-intervention.
